# A Mutation in the SCN1A Gene With a Peculiar Course: A Case Report

**DOI:** 10.7759/cureus.13612

**Published:** 2021-02-28

**Authors:** Lucia Sur, Gabriel Samasca, Genel Sur, Remus Gaga, Cornel Aldea

**Affiliations:** 1 Pediatrics, Iuliu Hatieganu University of Medicine and Pharmacy, Cluj-Napoca, ROU; 2 Immunology, Iuliu Hatieganu University of Medicine and Pharmacy, Cluj-Napoca, ROU; 3 Pediatrics, Emergency Clinical Hospital for Children, Cluj-Napoca, ROU

**Keywords:** epilepsy, febrile seizures, brain mri normal, child, no neurological degradation

## Abstract

In this report, we present the case of a one-year-old female patient with a history of febrile seizures, which was characterized by multiple seizures during hot baths and more than one episode of status epilepticus. Dravet syndrome was suspected due to the clinical context of the seizures and was confirmed by genetic testing. The brain MRI was found to be normal. Throughout the course of disease progression, the patient showed no signs of neurological degradation. The patient was found to have a mutation in the SCN1A gene with a peculiar course, which had not been reported previously. The normal psychomotor development, as seen in this case, highlights the different possibilities related to disease progression in Dravet syndrome.

## Introduction

The word epilepsy derives from the Greek word epilambanein, meaning repeated attacks. The prevalence of epilepsy in developed countries is around 0.5%. In the USA, nearly three million people have epilepsy, 450.000 being children under 17 years old. The International League Against Epilepsy has determined that for epilepsy to be considered drug-resistant, seizures need to persist even after two well-tolerated antiepileptic drugs have been administered in appropriate doses, either separately or as a combined therapy [1]. The most common cause of mortality in epilepsy and in status epilepticus is sudden unexpected death [2].

SCN1A encodes the α1 subunit of the sodium channel and is considered the most important epilepsy-related gene. There are a number of epilepsy phenotypes associated with the gene, the most commonly encountered being Dravet syndrome. Gene mutations are also associated with antiepileptic drug resistance.

Dravet syndrome is a severe type of myoclonic epilepsy that occurs in infants. It is a genetic disease with a low prevalence (between 1/15,000-1/40,000 infants). The clinical presentation involves myoclonic seizures that occur before one year of age, commonly associated with fever. Initially, the seizures are associated with alternating half-body laterality from one episode to the next. After the infant turns one, new types of seizures appear (atypical absence seizures, generalized tonic-clonic seizures, atonic and tonic seizures, and non-convulsive status epilepticus), and psychomotor decline is usually observed. The syndrome leads to developmental and epileptic encephalopathy (DEE).

## Case presentation

Our patient was a female infant who was one year and 10 months of age. The family history revealed a paternal uncle with febrile seizures and diabetes mellitus type I since age four; however, siblings and parents denied ever having similar symptoms. Personal history indicated that the patient was the second child, had regular development during pregnancy, the birth was through C-section at 38 weeks, weight at birth was 3,350 g, her APGAR score was 10, and she had no perinatal issues.

Starting when she was five-month-old, the patient had had multiple infections of the upper respiratory tract associated with febrile seizures, interpreted as common pediatric febrile seizures. At seven months old, the patient had the first seizure episode, which had been unrelated to fever or infection, during a hot bath. The patient had presented drifting of the eyes to the right, generalized hypotonia for approximately 10 minutes, with spontaneous remission, and postictal drowsiness.

Two weeks later, the patient had experienced the second seizure episode during a hot bath, a generalized tonic-clonic seizure with fixed eyes, loss of consciousness, and hypertonia of the right side of the body. The episode had lasted about 10-15 minutes. The patient had been taken to the ER, where the symptoms had remitted completely two minutes after receiving one dose of intra-rectal diazepam 5 mg/2.5 mL.

During the hospital stay, the patient was treated with 0.5 mL/day of phenobarbital 200 mg/2mL (usual dose: 6-10 mg/kg/dose). Because she presented status epilepticus associated with a fever of 39 °C, phenobarbital was replaced with 2 x 2 mL of sodium valproate 200 mg/5mL (usual dose: 15 mg/kg/day). But this caused abdominal pain in the patient, and she was started on 2 x 0.8 mL/day of levetiracetam 100 mg/mL (usual dose: 10 mg/kg/dose). Sodium valproate was eventually reintroduced without issues.

At home, the patient experienced multiple episodes of loss of consciousness, with right-side head deviation while sitting. A month after being discharged, she experienced the second episode of status epilepticus, once again with a fever of 39 °C, with right-side head and eye deviation, and left-side clonisms.

**Figure 1:**
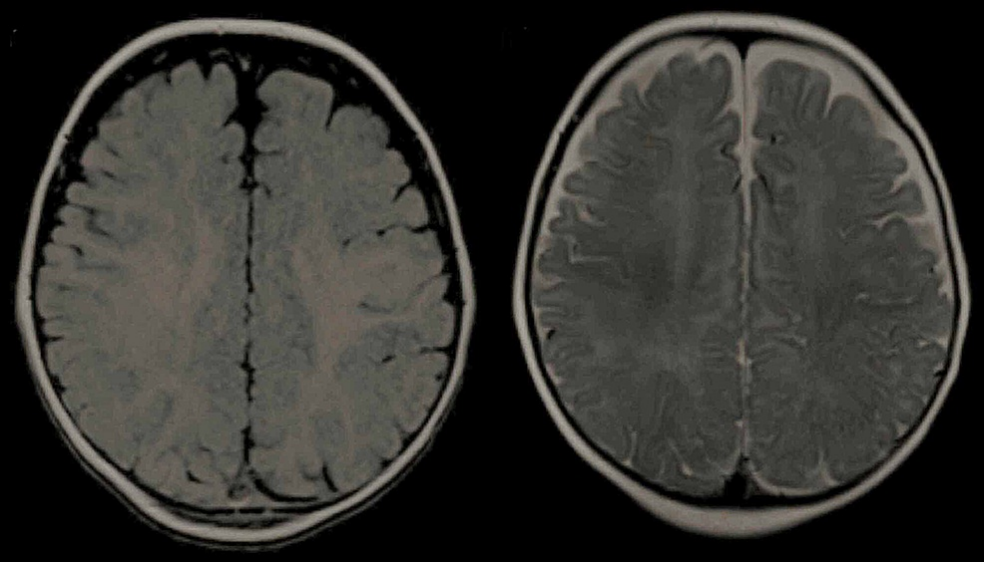
MRI in T1 and T2 sequences MRI: magnetic resonance imaging

Dravet syndrome was eventually suspected, and therapy with stiripentol 250 mg was started, with doses of 2 x 80 mg/day (usual dose: 20 mg/kg/day), in addition to sodium valproate. Levetiracetam therapy was discontinued, as it was considered insufficient after the second status epilepticus. The patient underwent genetic testing for epilepsy (chromosomal microarray), which revealed a mutation in the SCN1A gene. The brain MRI appeared normal (Figure 1). The physical exam was also normal except for slight hypotonia and bilateral genu recurvatum without psychomotor retardation.

## Discussion

This case was an interesting one because of the method of the onset, the difficulties that arose during the differential diagnosis, and the fact that no neurological degradation was detected. The majority of cases documented in the literature manifest severe psychomotor degradation, but there have also been rare cases with mild or normal development. Also, initially, the patient had received a relatively simple treatment scheme, which was gradually adapted to the increasing severity of the symptoms. The onset of symptoms before one year of age, the unsatisfactory response to antiepileptic medication, and the normal MRI (Figure 1) pointed towards Dravet syndrome. The diagnosis was confirmed by genetic testing, which showed SCN1A mutation. The primary diagnosis that was excluded was febrile seizures [3]. Although the patient initially presented febrile seizures, the symptoms had manifested during a hot bath, which, by increasing the bodily temperatures with no fever, had represented a triggering factor. Other excluded diagnoses included Lennox Gastaut syndrome [4], which has a later onset of symptoms and different seizure types, and, without going into detail, myoclonic atonic epilepsy, epilepsy of infancy with migrating focal seizures, early-onset SCN1A DEE, hemiplegic migraine, autism spectrum disorders, and non-epileptic SCN1A phenotype. The diagnosis was even more challenging to establish because seizures were not initially accompanied by fever. The seizures later being set off by slight elevation in body temperature and the fact that no psychomotor retardation was found summed up the case's peculiarity. The fact that no one else in the family had a documented case of Dravet syndrome can be possibly explained by de novo mutations, which are the most common forms found in the literature. One limitation of this case study was the fact that the patient was followed up at the Pediatric Neurology Clinic, and hence the case evolution was not sufficiently assessed. The new methods of treatment include pharmaceutical products such as fenfluramine, cannabidiol, and stiripentol (which was used in the current case as well).

## Conclusions

We presented the case of a patient with a mutation in the SCN1A gene with a peculiar course, which had not been reported previously. The normal psychomotor development that was observed sheds light on the different possibilities related to disease progression in Dravet syndrome.

## References

[1] Kwan P, Arzimanoglou A, Berg AT (2010). Definition of drug resistant epilepsy: consensus proposal by the ad hoc Task Force of the ILAE Commission on Therapeutic Strategies. Epilepsia.

[2] Swaiman KF (2012). Swaiman's Pediatric Neurology: Principles and Practice. Edinburgh: Elsevier Saunders.

[3] Skluzacek JV, Watts KP, Parsy O, Wical B, Camfield P (2011). Dravet syndrome and parent associations: the IDEA League experience with comorbid conditions, mortality, management, adaptation, and grief. Epilepsia.

[4] Cooper MS, Mcintosh A, Crompton DE (2016). Mortality in Dravet syndrome. Epilepsy Res.

